# Fine-mapping of a putative glutathione S-transferase (GST) gene responsible for yellow seed colour in flax (*Linum usitatissimum*)

**DOI:** 10.1186/s13104-022-05964-x

**Published:** 2022-02-20

**Authors:** Lester Young, Leonid Akhov, Manoj Kulkarni, Frank You, Helen Booker

**Affiliations:** 1 Department of Plant Sciences, University of Saskatchewan, 51 Campus Drive, Saskatoon, SK S7H 3R2 Canada; 2 National Research Council Saskatoon, 110 Gymnasium Place, Saskatoon, SK Canada; 3 Ottawa Research and Development Centre, Agriculture and Agri-Food Canada, 960 Carling Avenue, Ottawa, ON K1A 0C6 Canada; 4 Department of Plant Agriculture, University of Guelph, Crop Science Building, 50 Stone Road E, Guelph, ON N1G 2W1 Canada

**Keywords:** Flax, Yellow seed, Proanthocyanidin, Glutathione S-transferase

## Abstract

**Objective:**

The brown seed coat colour of flax (*Linum ustiatissimum*) results from proanthocyanidin synthesis and accumulation. Glutathione S-transferases (GSTs), such as the TT19 protein in *Arabidopsis*, have been implicated in the transport of anthocyanidins during the synthesis of the brown proanthocyanidins. This study fine mapped the *g* allele responsible for yellow seed colour in S95407 and identified it as a putative mutated GST.

**Results:**

We developed a Recombinant Inbred Line population with 320 lines descended from a cross between CDC Bethune (brown seed coat) and S95407 (yellow seed) and used molecular markers to fine map the *G* gene on Chromosome 6 (Chr 6). We used Next Generation Sequencing (NGS) to identify a putative GST was identified in this region and Sanger sequenced the gene from CDC Bethune, S95407 and other yellow seeded genotypes. The putative GST from S95407 had 13 SNPs encoding, including four non-synonymous amino acid changes, compared to the CDC Bethune reference sequence and the other genotypes. The GST encoded by Lus10019895 is a lambda-GST in contrast to the *Arabidopsis* TT19 which is a phi-GST.

**Supplementary Information:**

The online version contains supplementary material available at 10.1186/s13104-022-05964-x.

## Introduction

Flax (*Linum usitatissimum* L.) has brown seeds although some consumers prefer the yellow seeded varieties that exist. Polymeric proanthocyanidins (PA, or condensed tannins) are responsible for the brown seed coat colour in many species [1], including flax. Mutations in the genes of the PA biosynthetic pathway may result in yellow seed colour in flax, *Arabidopsis* and other species [2–6]. For example, in *Arabidopsis* a mutated glutathione synthase (GST), *tt19-1*, cannot transport the colourless anthocyanidin quercetin-3-O-rhamnoside across the tonoplast membrane and, consequently, accumulation of PA in the vacuole does not occur [2, 7]. In flax five gene alleles (*Y*, *b1*, *b1*^*vg*^, *d* and *g*), each individually responsible for yellow (or mottled) seed colour, have been observed and their genetics partially elucidated [8], however, the functional and genetic identity of some of these genes has only recently been studied. The location and identity of the mutated *D* gene in cultivar Bolley Golden was determined to be a flavonoid 3′5′ hydroxylase on Chr2 [5, 6], and the dominant *Y* gene was found to be due to insertion of a transposon upstream of chalcone synthase (unpublished data). The mutated *G* gene was selected for fine mapping as it is one of the remaining known yellow seed coat coloured mutants and thought to be a single gene. It is not known if the *b1* and *b1*^*vg*^ mutants are different genes or allelic.

Flax has a haploid number of 15 and a genome size of ~ 380 Mbp. The reference sequence from CDC Bethune, was published first as scaffolds [9] and, more recently, as pseudomolecules [10]. Genome-wide molecular markers covering the entire genome are available [11, 12].

Our objective was to fine map the *G* gene in flax using the yellow seed line S95407 developed at the University of Saskatchewan. Characterizing the *g* gene could assist breeding cultivars of yellow seeded flax.

### Main text

#### Material and methods

A detailed description of the materials and methods used are available as Additional file 1 (which contains references [14] and [19]).

#### Results and discussion

**Fig. 1:**
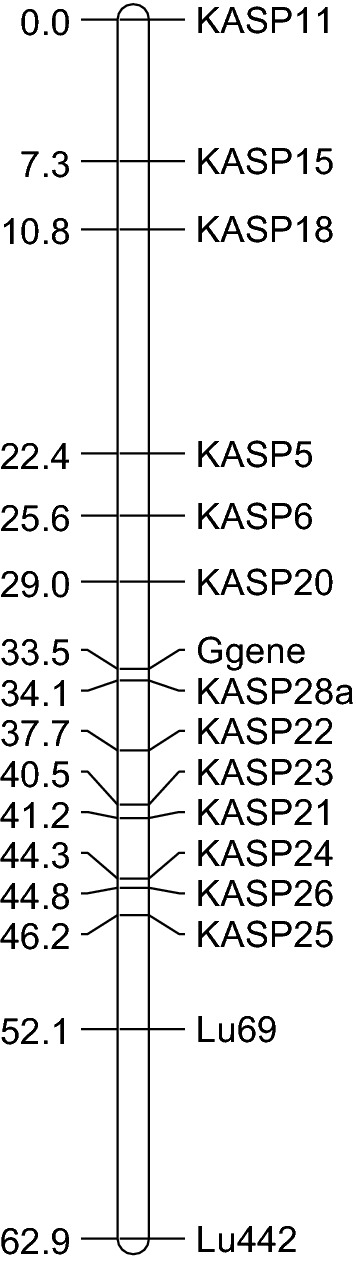
Map of molecular markers and putative *G* gene location in LG6. Initial mapping with SSR markers placed Lu69 and Lu442 close to the putative *G* gene (not shown). Distances, in cM, were determined using Kosambi’s mapping function in JoinMap 4.1

We mapped the location of the G gene first using Simple Sequence Repeat (SSR) markers and then performed fine mapping of the locus using Kompetetive Allele Specific PCR (KASP) markers. Initial analysis of the 193 SSR markers [13] indicated that 123 were polymorphic between CDC Bethune and S95407. Testing these polymorphic markers on pooled DNA from a subset of 10 brown seeded or 10 yellow seeded individuals identified 52 markers with an unequal distribution of alleles. Thirty of these markers, selected based on their distribution over the 15 flax chromosomes, were used to screen a subset of 94 individuals and the two parents (Additional file 4: Data S1). We determined that marker Lu442, on Chr6, was located  ~ 30 cM from the *G* gene. Six other polymorphic markers on Chr6 were then used to screen the population, revealing that Lu69 was located ~ 20 cM the *G* gene (Fig. 1, Table 1 and Additional file 4: Data S1). Illumina HiSeq was used to resequence S95407 (archived at NCBI Sequence Read Archive SRR11869873), the reads trimmed using *trimmomatic* [15] and aligned against the CDC Bethune reference sequence [9] using *bowtie2 *[16]. Refinement of the alignment, variant calling and filtering SNPs between S95407 and CDC Bethune was performed using *samtools *[17]and *bcftools *[18]*.* The script used to identify SNPs is available in the Additional file 1. KASP markers (KASP1-18) were designed against SNPs located distally from Lu69 in the region Chr6:11.65–17.86 Mbp. Lu69 is located at Chr6:10.96 Mbp. Markers KASP5 and KASP6 were 11.1 and 7.9 cM from the *G* gene, or at Chr6:15.07 Mbp and Chr6:14.84 Mbp, respectively (Fig. 1, Table 1 and Additional file 4: Data S1).

**Table 1:** Molecular markers associated with *G* gene for seed coat colour in flax

Marker	Primer sequence	Scaf-fold number	Physical location on scaffold (bp)	Physical location Chr 6 (bp)	Genetic distance from *G* gene (cM)
Lu442	F: TCCGTGTAGAAGAAACGAGGA R: CCGACCTCTTGCCATGATTA	25	1,636,327		29.4
Lu69	F: CTAAACCACACCCCCATCAC R: AAAGTGGGGAAATTGGGCTA	352	68,959–69,164	10,960,833–10,961,358	18.6
KASP5	A1: GTTCAAGCTTCCTAAGCAGGCG A2: GGTTCAAGCTTCCTAAGCAGGCA C1: GGTGGTTAGATTCCTGGCCGGAA	176	258,853–258,911	15,065,122–15,065,180	11.1
KASP6	A1: ATCTGTAATCTAACGTCCGAGCAGT A2: GATCTGTAATCTAACGTCCGAGCAAC C1: CCATACAAAATCTCAATTCGACGCTTCTA	176	485,174–485,241	14,838,792–14,838,886	7.9
KASP20	A1: CTCCGTTTCATTATAGAATTGCTGGATTCA A2: CTTCGTTTCATTATAGAATTGCTGGATTCT C1: CTAGCACAAAATTAACCAGACTATGTAGA	1491	5180–5380	14,320,238–14,320,179	4.5
KASP28	A1: ACGATCGAAAGAGGAAGCTCG A2: ACGATCGAAAGAGGAAGCTCA C1: TACATGCATATGGCTAGCTACACTT	1491	501,848–501,870	13,793,920–13,794,025	0.6
KASP22	A1: GGTAGCTAGAGTTAGCTCATGATGA A2: GTAGCTAGAGTTAGCTCATGATGG C1: CTACTATCACATAGCTTTGAGCTACACTT	618	47,530–47,737	13,450,328–13,450,385	4.2
KASP21	A1: ACCCAATCAGCTCTGGATTTGTTTAA A2: ACCCAATCAGCTCTGGATTTGTTTAT C1: CTTGCCAGAAATCCAATACCGTCTCAA	618	538,471–538,670	12,959,816–12,959,880	7.7
HRM LG6:13,446	F: CTCTGCTTTACTACTCTTTACTCT R: AGTTCTAATATGAAATCAGAGGGG			13,446,488–13,446,617	
HRM LG6:13,593	F: TGACACTATTGTAGGATTTGTTTCT R: CGTCGAATTAATCTCTACCTGAAT			13,593,300–13,593,430	
HRM LG6:13,937	F: ACAGAGAAACATCAATCGAAATCT R: CATTTGCTTCGGCTTATTTGC			13,936,796–13,936,923	
HRM LG6:13,942	F: GAAGGTATTGGAAATGGACCATAT R: TACAAGAAAAGCAGAGCAACTTAT			13,941,869–13,941,995	
HRM LG6:14,010	F: CTTGCCCATTTCCTCCATTATTTT R: AAGATTAGAAATCAAAGTACGGCA			14,009,768–14,009,893	

Scaffold number and location are based on reference genome sequence version 1.0 (available at phytozome-next.jgi.doe.gov/info/Lusitatissimum_v1_0). Physical coordinates on Chr 6 are based on pseudomolecule sequence CP027630.1 in NCBI. The putative *G* gene is located at 13,779,760–13,782,089 of Chr 6. Genetic distances between markers and *G* gene were determined using Kosambi’s mapping function

*F* forward primer; *R* reverse primer; *A1* allele specific primer 1 *A2* allele specific primer 1 *C1* common primer

Markers spanning the region between KASP6 and Lu69 were developed (KASP 19–27) and mapped. KASP20 (on scaffold1491), KASP22 and KASP23 (both on scaffold618) were located approximately 4.5, 3.2 and 7.0 cM from the *G* gene, respectively (Table 1, scaffold information from phytozome-next.jgi.doe.gov/info/Lusitatissimum_v1_0). An additional marker approximately mid-way between KASP20 and KASP22 (KASP28) was developed to differentiate an SNP located ~ 250 kb from the distal end of scaffold1491. An additional 94 lines from the RIL were used to map the interval between KASP28 and the putative *G* gene (Additional file 4: Data S1). The S95407 allele for KASP28 segregated with all the 94 yellow seeded lines and only one of the 94 brown seed coat lines. Five High Resolution Melt (HRM) markers within 5 cM of the putative *G* gene (Table 1) were used to genotype the single brown seeded line with the yellow genotype. This individual was observed to have the yellow genotype for all five markers, indicating that it had been incorrectly phenotyped as a brown-seeded line.

Putative genes in the last 250 kb of scaffold1491 were identified from the CDC Bethune reference genome. This region corresponds to Chr6:13.5–13.8 Mbp, based on the pseudomolecule sequence published by You et al. [10]. This region contains 55 putative genes, of which 28 had one or more SNPs in the coding sequences between CDC Bethune and S95407. This region also contained the KASP28 marker and was adjacent to scaffold618, which contained the KASP22 marker. A portion of one gene (Lus10019895) in this region, located 15 kb from KASP 28 was a putative glutathione S-transferase (GST), as identified using TBLASTX. GSTs play a role in transporting anthocyanins or proanthocyanidin in many tissues, including the seed coat [2, 4, 20]. Lus10019895 was located between Chr6:13.8–13.8 Mbp, based on the flax pseudomolecule sequences.

The last six exons of the putative gene Lus10019895 encode for a GST, with the first 14 exons encode a putative thylakoid integral membrane TerC protein (Additional file 2: Figure S1). The putative TerC protein shares 80% amino acid residue similarity with the Arabidopsis TerC The GST encoded by the last six exons of Lus10019895 is 1185 bp long, encoding a 738 bp CDS.

**Fig. 2:**
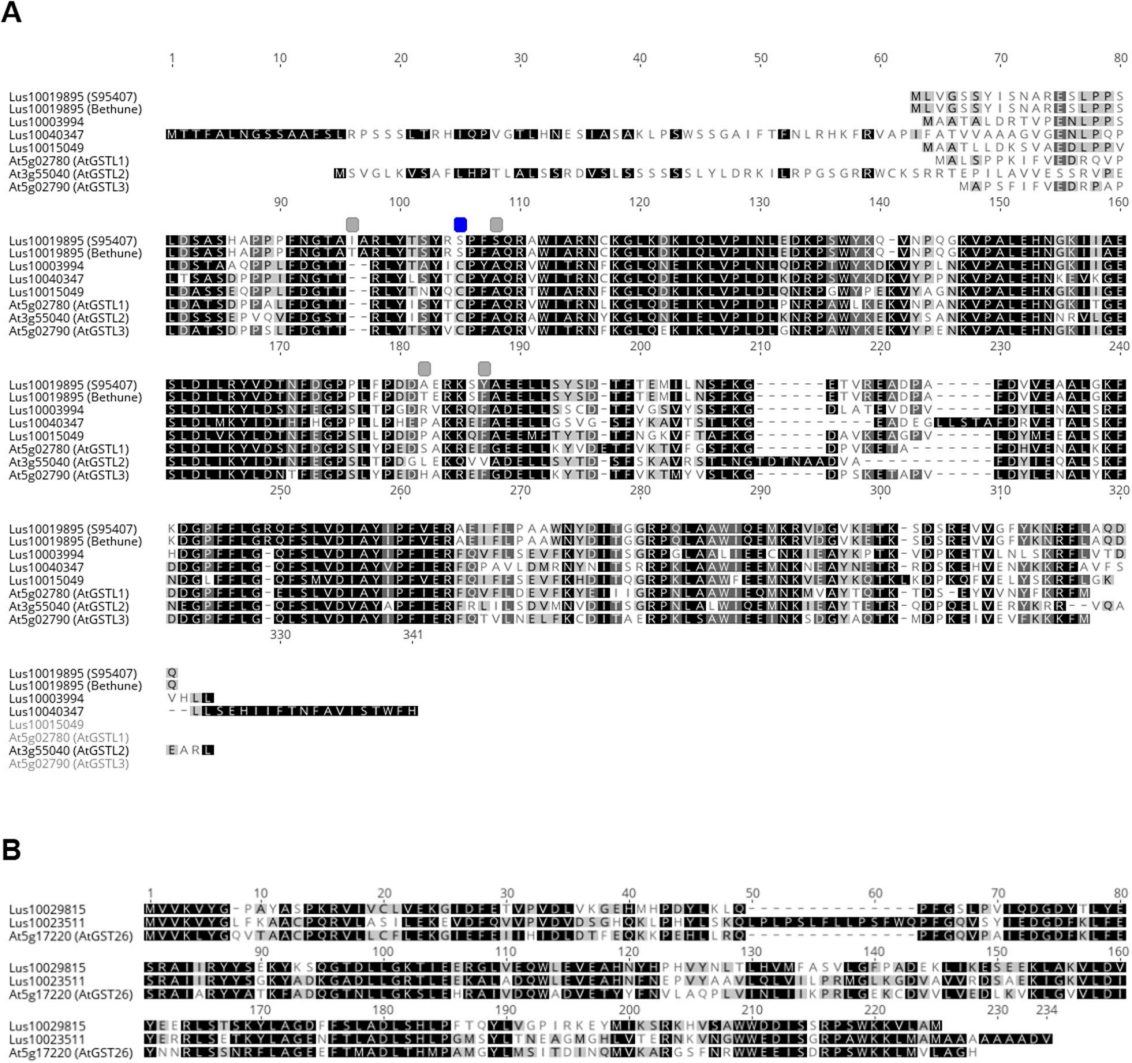
Alignment of putative Lus10019895 GST protein with some flax and *Arabidopsis* GST homologs. The putative Lus10019895 GST protein shares 70–74% similarity to the *Arabidopsis* lambda GSTs. Darker boxes around the amino acid residues indicate a higher consensus level at that position, based on amino acid similarity. **A** Alignment of both the putative CDC Bethune and S95407 Lus10019895 GST proteins with *Arabidopsis* lambda GSTs. Differences between the CDC Bethune and S95407 proteins are indicated with grey boxes above the sequences. The serine residue replacing the conserved cysteine in the active site of other GSTs is indicated with a blue box above the sequences. **B** Alignment of two flax putative Gamma GST proteins with the TT19 protein (At5g17220 AtGST26)

The sequence of the GST portion of Lus10019895 was determined by PCR amplifying this fragment from genomic DNA from brown seeded CDC Bethune and CDC Sanctuary and from yellow seeded, S95407, M96006 (*B1*^*vg*^ gene), Crystal (*B1* gene), G1186 (*D* gene) and YSED18 (*Y* gene) and then Sanger sequenced. The sequence of the PCR fragments were identical to the CDC Bethune reference sequence for all the genotypes except S95407 (See Additional file 5: Data S2). This data confirms the consensus sequence of Lus10019895 obtained from the S95407 NGS data obtained in this project. In the S95407, 13 SNPs were observed. Two SNPs were located in the 5′ UTR of the gene, two in the 3′UTR and three in proposed introns. A total of six SNPs were observed in CDS sequences, four of which were non-synonymous (Fig. 2A). These amino acid changes were T34I, A46S, T121A and F126Y. The conformation of the active site in the S95407 Lus10019895 GST may be disrupted by the A46S change, as this alanine is highly conserved, and/or the T34I substitution. The A46S change in S95407 may be particularly significant as it may result in significant alteration in the electrochemical conformation of the active site. An alternative explanation for the yellow seeded phenotype observed in S95407 is a reduction in Lus10019895 expression brought about by a 24 bp deletion in the 3′UTR, 658 bp downstream from the stop codon (not shown).

In the developing seed coat GSTs are thought to transfer glutathione onto anthocyanins or PA prior to transport into the vacuole. A GST mutant, *tt19*, is associated with the development of yellow seeds in *Arabidopsis* [2]. GSTs are involved in the transport of anthocyanins and PA in the seed coat of grape [20]. Homologues of *TT19* are involved in the transport of anthocyanins in the petals of cyclamen [21] and petunia [22]. The Lus10019895 GST shares share 71.7%, 74.2% and 66.0% similarity to three homologs from flax, Lus10003994, Lus10015049 and Lus10040347, respectively. Collectively, these genes share 67–71% similarity at the amino acid level to the *Arabidopsis* lambda-type GST proteins *AtGSTL1*, *AtGSTL2* and *AtGSTL3*) (Fig. 2A), but only 19% identity and 33–37% similarity to *AtGST26*/*TT19*/*AtGST phi12* (not shown). Three other flax GST proteins, Lus10023511, Lus10029815 and Lus10040393, had a much higher degree of similarity to AtGST26/*TT19* (66%, 68% and 72%, respectively) (Fig. 2B).

Both lambda-GSTs and phi-GSTs are expressed in the seeds of *Brassica napus* [23], *Vitus vinifera* [20], *Helianthus annuus* [24] and *Capsicum annuum* [25]. Anthocyanin transport into the vacuole is facilitated by multiple classes of GSTs in maize [26]. Three out of four grape GSTs examined complement the function *tt19* in *Arabidopsis*, albeit in different ways [20], so it is plausible that the Lus10019895 GST performs this function in maturing flaxseed, despite having less homology to AtGST26 than other GST homologues in flax. Interestingly, the Lus10019895 protein lacked the highly conserved cysteine at residue 43, in the active site of both lambda- and phi-type GSTs and had a serine instead (Fig. 2). The other flax GST proteins, except Lus10029815, still retained the cysteine at this site. Lus10019895 is more similar to non-lambda GSTs from other species (Additional file 3: Figure S2), which often have a serine residue rather than a cysteine at this position in the active site [27], than to phi-GSTs in other species [20, 23–25, 27]. The Lus10019895 GST protein has 76–78% similarity to the *Citrus sinensis* (XP006480546), *Eucalyptus grandis* (XP010047051), and *Jatropha curcas* (NP001295698) GSTs and shares a high degree of similarity with homologs from other species (Additional file 3: Figure S2). The Lus10019895 protein shares only 37% similarity with the petunia phi-type GST responsible for anthocyanin transport in petals, AN9 [22].

A BLAST search of flax ESTs in NCBI using the Lus10019895 CDS returned 10 hits, all from the mature embryo EST library (LIBEST_027001). The consensus sequences of both CDC Bethune and S95407 around Lus10019895 are provided in Additional file 5: Data S2.

#### Summary

We have identified, using molecular markers, bioinformatics and DNA sequencing, a putative GST involved in PA synthesis in the seed coat of flax. The putative GST is encoded in the last six codons of *Lus10019895* which appears to be artefactually fused to a TerC gene. As many as 13 SNPs, including four non-synonymous changes, are observed in the yellow-seed coat coloured mutant, S95407, compared to the brown-seed coat coloured reference sequence from CDC Bethune. The *Lus10019895* GST has a higher level of similarity to Lambda-type GSTs from *Arabidopsis* and other species than to phi-type GSTs such as the *Arabidopsis TT19* and Petunia *AN9*.

#### Limitations

The observation that *Lus10019895* consists of two genes could be proven definitively using RT-qPCR, however, we assume that the TerC and GST genes are separate based on the arrangement of CDS and high level of similarity to homologs within the flax genome. We do not determine that the putative GST identified here is functionally responsible for brown seed coat colour in CDC Bethune, or that the mutant gene is the cause of the yellow seed coat colour in S95407.

## Supplementary Information

**Additional file 1: **Detailed materials and methods.

**Additional file 2: Figure S1.** Putative CDS structure of *Lus10019895* and alignment with *Arabidopsis* TerC and GST proteins. **A** Lus10019895 is 4467 bp long and contains 20 CDS (yellow arrows). The first 14 CDS of the gene code for a thylakoid membrane protein, TerC, while the last six exons code for a Glutatione-S transferase. Coloured boxes indicate identical amino acid residues. **B** Alignment of *Arabidopsis* TerC (XP020876262) and the first 14 putative exons in Lus10019895 with 80% amino acid similarity. **C** Alignment of Arabidopsis GST protein At5g02780 and the last six CDS of Lus10019895, showing 75% amino acid similarity.

**Additional file 3: Figure S2.** Alignment of *Lus10019895* protein with GST proteins from other species. Darker shading of residue background indicates a greater number of similar residues at that position. Rectangular boxes indicate non-synonymous changes in amino acid residues between S95407 and CDC Bethune proteins. Dendrogram indicates relatedness of the GST proteins.* Lus10019895* from *L. usitatissimum* has greater similarity to the *Arabidopsis* lambda GSTs than to AtGST26 (TT19) from *Arabidopsis*.

**Additional file 4: Data S1.** Markers and genotypes of S95407 × CDC Bethune RIL population segregating for yellow seed coat colour. The first 94 lines in the population were phenotyped using the SSR markers (Lu69 and Lu442) and KASP markers (KASP5-26). These lines plus an additionaly 94 lines were genotyped using KASP28. Phenotype a = yellow seed coat colour, b = brown seed coat colour. For genotype data h = heterozygote and – = missing data.

**Additional file 5: Data S2.** Sequences of *Lus10019895* for CDC Bethune and S95407.

## Data Availability

All data generated or analysed during this study are included in this published article and its Additional files. Resequencing data from S95407 is available at NCBI SRA SRR11869873.

## Abbreviations

GST: Glutathione S-transferase
HRM: High-resolution melt
KASP: Kompetitive allele specific PCR
LG: Linkage group
NGS: Next generation sequencing
PA: Proanthocyanidin
RIL: Recombinant inbred line
SSR: Simple sequence repeat
