# Supplementary material for: Fine-mapping of a putative glutathione S-transferase (GST) gene responsible for yellow seed colour in flax (Linum usitatissimum)
Source: BMC Res Notes. 2022 Feb 20;15:72. doi: 10.1186/s13104-022-05964-x (PMC8859895; doi:10.1186/s13104-022-05964-x)
Supplement: Supplementary file 1 — Additional file 1: Detailed materials and methods. [file 13104_2022_5964_MOESM1_ESM.docx]

**Additional file: Detailed Materials and Methods**

*RIL population*

A RIL population with 320 individual lines was developed to the F6 generation via single seed descent from a cross of CDC Bethune (brown seed coat) x S95407 (yellow seed coat colour). Seed colour of each line was determined visually.

*Molecular marker mapping and screening*

Bulked segregant analysis, to determine if the 193 previously developed SSR markers (13) were polymorphic, was performed on DNA pooled from 10 randomly selected brown seeded and 10 randomly selected yellow seeded individuals from the RIL population. Briefly, individual marker reactions were amplified and labelled with one of four fluorescent dyes from highly purified genomic DNA (Qiagen 96 DNA Easy Plant Kit) of individual plants. The individual SSR reactions were multiplexed with up to four different fluorophores/SSRs in each well. Separation of fragments and size determination were performed using capillary electrophoresis (Applied Biosystems 3130 Genetic Analyzer).

An initial genetic map using 30 of the 52 polymorphic markers was developed using 94 individual lines (47 brown seeded and 47 yellow seeded lines) along with the two parents. These markers were selected based on their positions on the 15 flax chromosomes. Two polymorphic SSR markers on Chr6, Lu442 and Lu69 were linked to the G gene. Map construction was developed using Join Map 4.1 (Kyazma, Netherlands).

Further mapping was performed on the 94 RIL lines and two parents using Kompetetive Allele Specific PCR (KASP) markers. The KASP1-18 markers were designed to differentiate between S95407 and CDC Bethune SNPs (Table 1. See SNP identification, below). These markers were located in the 5 Mbp distal to marker Lu69 on Chr6. Additional markers (KASP19-27) were developed to map the interval between Lu69 and KASP6 and identified scaffolds from the reference sequence located in this region of the genome. The KASP28 marker was developed, based on this refined map, and was mapped to 0.6 cM from the G gene using 188 lines (including the initial 94 lines). KASP28 and the G gene are co-located on scaffold1491 (sequence available at https://phytozome-next.jgi.doe.gov/info/Lusitatissimum_v1_0).

A single brown seeded individual had the yellow-seeded KASP28 allele. An additional five High Resolution Melt (HRM) markers spanning a 5 cM region (~560 kbp) around the putative G gene were developed to further genotype this individual. DNA for the KASP and HRM assays was extracted from frozen leaves using a rapid alkaline lysis method (14).

*SNP identification*

Next Generation Sequencing (Illumina HiSeq), performed by the National Research Council – Saskatoon sequencing facility, was used to resequence the recessive yellow seed parent S95407. Paired-end reads of 100 bp were generated and are available at NBCI’s Sequence Read Archive SRR11869873. Read files were transferred to a Compute Canada server for further processing and alignment. Briefly, the reads were trimmed and quality filtering using *trimmomatic* (15), aligned against the flax reference genome (CDC Bethune), version 1.0 (9) using *bowtie2* (16). Alignments were adjusted, normalized, fixed and filtered and SNPs called using *samtools* (17) and bcftools (18). The script used to perform these actions is included in this document. Default parameters were used for all these applications. The read alignments and SNPs were visualized and consensus sequences generated using Geneious 9.0 (Biomatters Inc, Auckland, New Zealand). This software was also used to design primers for the KASP and HRM assays (LGC group, Middlesex, UK), perform protein sequence alignment and phylogenetic analysis.

Analysis of the putative G gene

The putative GST gene was PCR amplified from genomic DNA from yellow-seeded S95407, M96006, Crystal, G1186, YSED and brown-seeded CDC Bethune and CDC Sanctuary using the proof-reading DNA polymerase Phusion (NEB). The PCR and internal sequencing primers were designed using Geneious against conserved sequences flanking the putative GST gene. Sanger sequencing of the PCR fragments, performed by NRC-Saskatoon’s sequencing facility. Alignment of the sequences was performed using Geneious.

Bowtie2 and the reference sequence of Lus10019895 were used to extract reads from the S95407 resequencing data. Reads aligning to Lus10019895 were assembled into a contig using the “Map to reference” function in Geneious. These results giave both a consensus sequence for the S95407 gene, as well as variants compared to the reference sequence from CDC Bethune.

#!/bin/bash

#SBATCH --time=12:00:00

#SBATCH --account=def-btaran

#SBATCH --job-name=FASTQ-to-bcf

#SBATCH --output=%x-%j.out

#SBATCH --cpus-per-task=8 # number of threads

#SBATCH --mem-per-cpu=6G # memory; default unit is megabytes

#Takes trimmed+filtered fastq files and aligns them against the supplied reference sequence

module load trimmomatic

module load bowtie2

module load samtools

#Set reference sequence. Change infiles1 and 2 to fastq readfiless

infile1="S0006F4_ACTTGA_L007_R1.fq.gz"

infile2="S0006F4_ACTTGA_L007_R2.fq.gz"

ref_seq="Bethune_pseudomolecules"

ref_seq_file="Bethune_pseudomolecules.fa"

basename="S95407_reads"

fixedbamfile=${basename}.fixmate.bam

printf "Using Trimmomatic on readfiles\n"

java -jar $EBROOTTRIMMOMATIC/trimmomatic-0.39.jar PE -threads 8 \

${infile1} ${infile2} -baseout ${basename}.fq.gz \

LEADING:5 SLIDINGWINDOW:4:15 MINLEN:80

#trimmomatic will gzip trimmed data in $basename_1P.fq.gz format

printf "Aligning ${basename} readfiles to ${ref_seq} using $infile1 and $infile2\n"

bowtie2 -p 8 --no-unal --no-mixed --no-discordant \

--rg-id ${basename} --rg SM:S95407 \

-x ${ref_seq} \

-1 ${basename}_1P.fq.gz -2 ${basename}_2P.fq.gz | \

samtools view -@ 8 -Shb | samtools sort -@ 8 -u | \

samtools view -@ 8 -b --write-index -h -o ${basename}.bam

printf "Fixmate and remove duplicates on $bamname \n"

samtools collate -@ 8 -u ${basename}.bam -O | \

samtools fixmate -@ 8 -r -u -m - - | \

samtools sort -@ 8 -u -T tempfile - | \

samtools markdup -@ 8 -r --reference ${ref_seq_file} - ${fixedbamfile}

#This script does mpileup and calls SNPs in alignments from multiple Bison and Novelty readfiles

bcftools mpileup --threads 8 -C 50 -O u -a AD,DP,ADF,ADR \

-f ${ref_seq_file} ${fixedbamfile} | \

#Adjusting MQ using -C 50. Including DP and AD tags.

bcftools norm --threads 8 -c s -O u -f ${ref_seq_file} | \

#split multiallelic sites into a single biallelic one using -m1.

#Does only SNPs. Fix bad sites (in ref seq?) using -c s

bcftools call --threads 8 -m -v -G- -A -V indels -O b -o ${basename}.bcf

#each sample is called individually and no HWE called (using -G-).

#Only variants called (-v). Output bcf

#include all alt alleles with -A. Skip indels (-V indels)
