# Supplementary material for: Fine-mapping of a putative glutathione S-transferase (GST) gene responsible for yellow seed colour in flax (Linum usitatissimum)
Source: BMC Res Notes. 2022 Feb 20;15:72. doi: 10.1186/s13104-022-05964-x (PMC8859895; doi:10.1186/s13104-022-05964-x)
Supplement: Supplementary file 2 — Additional file 2: Figure S1. Putative CDS structure of Lus10019895 and alignment with Arabidopsis TerC and GST proteins. A Lus10019895 is 4467 bp long and contains 20 CDS (yellow arrows). The first 14 CDS of the gene code for a thylakoid membrane protein, TerC, while the last six exons code for a Glutatione-S transferase. Coloured boxes indicate identical amino acid residues. B Alignment of Arabidopsis TerC (XP020876262) and the first 14 putative exons in Lus10019895 with 80% amino acid similarity. C Alignment of Arabidopsis GST protein At5g02780 and the last six CDS of Lus10019895, showing 75% amino acid similarity. [file 13104_2022_5964_MOESM2_ESM.docx]

**Figure S1**

A


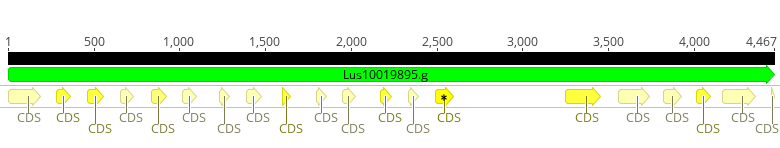


B


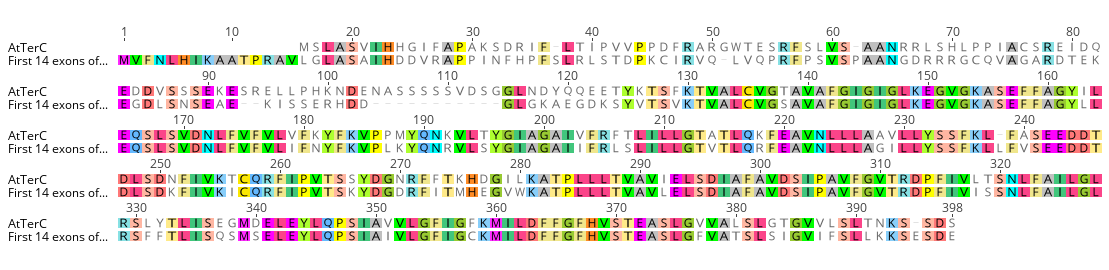


C


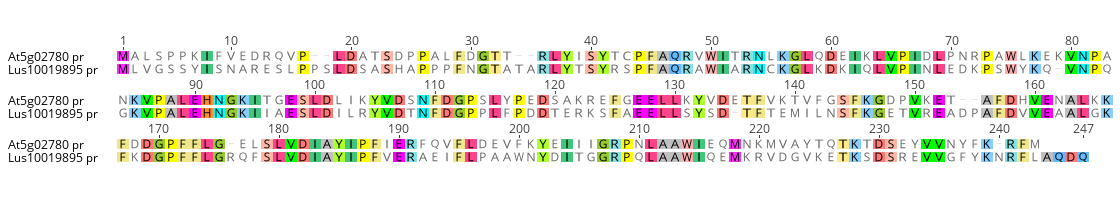


Figure S1: Putative CDS structure of Lus10019895 and alignment with Arabidopsis TerC and GST proteins. A) Lus10019895 is 4467 bp long and contains 20 CDS (yellow arrows). The first 14 CDS of the gene code for a thylakoid membrane protein, TerC, while the last six exons code for a Glutatione-S transferase. Coloured boxes indicate identical amino acid residues. B) Alignment of Arabidopsis TerC (XP020876262) and the first 14 putative exons in Lus10019895 with 80% amino acid similarity. B) Alignment of Arabidopsis GST protein At5g02780 and the last six CDS of Lus10019895, showing 75% amino acid similarity
