# Supplementary material for: Fine-mapping of a putative glutathione S-transferase (GST) gene responsible for yellow seed colour in flax (Linum usitatissimum)
Source: BMC Res Notes. 2022 Feb 20;15:72. doi: 10.1186/s13104-022-05964-x (PMC8859895; doi:10.1186/s13104-022-05964-x)
Supplement: Supplementary file 3 — Additional file 3: Figure S2. Alignment of Lus10019895 protein with GST proteins from other species. Darker shading of residue background indicates a greater number of similar residues at that position. Rectangular boxes indicate non-synonymous changes in amino acid residues between S95407 and CDC Bethune proteins. Dendrogram indicates relatedness of the GST proteins. Lus10019895 from L. usitatissimum has greater similarity to the Arabidopsis lambda GSTs than to AtGST26 (TT19) from Arabidopsis. [file 13104_2022_5964_MOESM3_ESM.docx]

**­­­**

**_
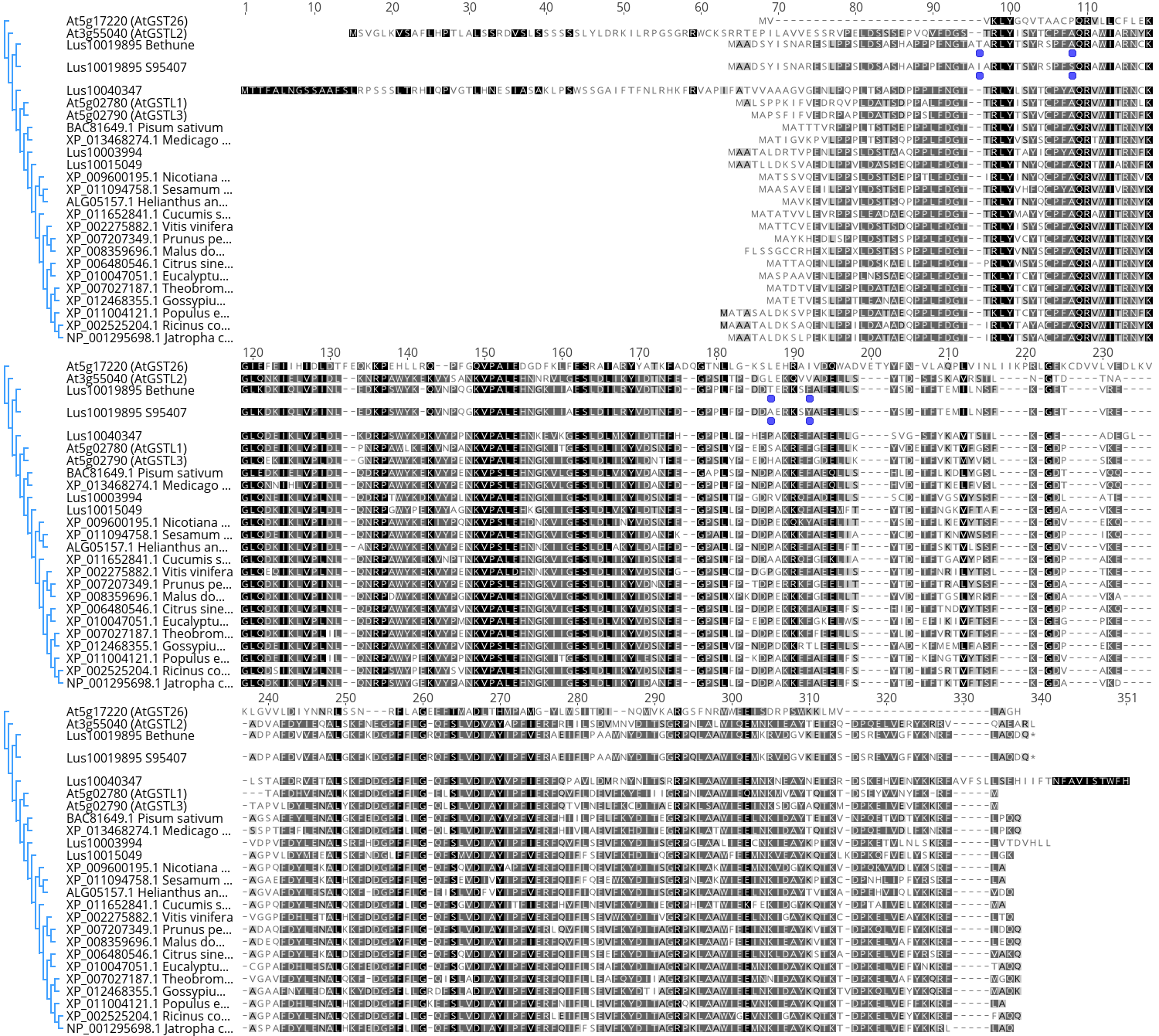
_Fig. S2** Alignment of *Lus10019895* protein with GST proteins from other species. Darker shading of residue background indicates a greater number of similar residues at that position. Rectangular boxes indicate non-synonymous changes in amino acid residues between S95407 and CDC Bethune proteins. Dendrogram indicates relatedness of the GST proteins. *Lus10019895* from *L. usitatissimum* has greater similarity to the *Arabidopsis* lambda GSTs than to AtGST26 (TT19) from *Arabidopsis*
