# Supplementary material for: Fine-mapping of a putative glutathione S-transferase (GST) gene responsible for yellow seed colour in flax (Linum usitatissimum)
Source: BMC Res Notes. 2022 Feb 20;15:72. doi: 10.1186/s13104-022-05964-x (PMC8859895; doi:10.1186/s13104-022-05964-x)
Supplement: Supplementary file 4 — Additional file 4: Data S1. Markers and genotypes of S95407 × CDC Bethune RIL population segregating for yellow seed coat colour. The first 94 lines in the population were phenotyped using the SSR markers (Lu69 and Lu442) and KASP markers (KASP5-26). These lines plus an additionaly 94 lines were genotyped using KASP28. Phenotype a = yellow seed coat colour, b = brown seed coat colour. For genotype data h = heterozygote and – = missing data. [file 13104_2022_5964_MOESM4_ESM.docx]

Data S1: Markers and genotypes of S95407 x CDC Bethune RIL population segregating for yellow seed coat colour. The first 94 lines in the population were phenotyped using the SSR markers (Lu69 and Lu442) and KASP markers (KASP5-26). These lines plus an additionaly 94 lines were genotyped using KASP28. Phenotype a = yellow seed coat colour, b = brown seed coat colour. For genotype data h = heterozygote and - = missing data.

| Line | KASP14 | KASP5 | KASP6 | KASP7 | KASP10 | KASP11 | KASP15 | KASP18 | KASP20 | KASP21 | KASP22 | KASP23 | KASP24 | KASP25 | KASP26 | Lu69 | Lu442 | KASP28 | Pheno-type |
| --- | --- | --- | --- | --- | --- | --- | --- | --- | --- | --- | --- | --- | --- | --- | --- | --- | --- | --- | --- |
| RIL105 | B | B | B | B | B | A | A | H | B | B | B | B | B | B | B | B | B | B | b |
| RIL106 | B | B | B | B | B | B | B | B | B | B | B | B | B | B | B | B | B | B | b |
| RIL107 | B | B | B | A | B | B | B | B | B | B | B | B | B | B | B | B | B | B | b |
| RIL108 | H | A | A | A | A | A | A | A | A | A | A | B | B | B | B | B | B | A | a |
| RIL110 | A | B | B | B | A | A | A | A | B | B | A | B | B | B | B | B | B | A | a |
| RIL111 | A | A | A | A | A | B | B | B | A | A | A | A | A | A | A | A | A | A | a |
| RIL112 | B | B | A | B | B | A | A | A | A | A | A | A | A | A | A | A | A | A | a |
| RIL113 | B | A | - | B | A | A | A | A | A | A | A | A | A | A | A | A | A | A | a |
| RIL114 | A | B | B | B | A | B | B | B | B | B | B | B | B | B | B | B | B | B | b |
| RIL115 | A | A | A | B | A | B | B | B | A | A | A | A | A | H | A | H | B | A | a |
| RIL116 | B | B | B | A | A | B | B | B | B | B | B | B | B | B | B | B | B | B | b |
| RIL117 | B | B | B | B | B | A | A | A | B | B | B | B | B | B | B | H | B | B | b |
| RIL118 | B | A | A | A | B | A | A | A | A | B | B | B | B | B | B | B | B | A | a |
| RIL119 | A | A | A | B | B | A | A | A | A | A | A | A | A | A | A | A | A | A | a |
| RIL120 | A | A | A | B | A | A | A | A | A | A | A | A | A | A | A | A | B | A | a |
| RIL121 | A | A | A | A | B | A | A | A | A | A | A | A | B | B | B | B | B | A | a |
| RIL122 | B | B | B | B | A | A | B | B | B | B | B | B | B | B | B | B | B | B | b |
| RIL123 | A | B | B | B | B | B | B | B | A | A | A | A | A | A | A | A | A | - | a |
| RIL124 | B | B | B | B | A | B | B | B | B | B | B | B | B | A | B | H | A | A | a |
| RIL125 | A | A | A | B | B | A | A | A | B | B | B | B | B | B | B | B | B | B | b |
| RIL126 | A | B | B | B | B | B | B | B | B | B | B | B | B | B | B | B | B | B | b |
| RIL127 | B | B | B | A | A | A | A | A | B | A | A | A | A | A | A | A | A | B | b |
| RIL128 | B | A | A | B | B | A | A | A | A | A | A | A | B | B | B | B | A | **B** | **a** |
| RIL129 | A | H | A | B | B | A | A | A | A | H | A | A | H | A | H | B | H | A | a |
| RIL131 | B | A | A | A | B | B | A | H | A | A | A | A | A | A | A | A | A | - | a |
| RIL132 | B | A | A | B | B | A | A | A | A | B | A | B | B | B | B | B | B | A | a |
| RIL133 | A | A | A | B | A | A | A | A | A | A | A | A | A | A | A | A | A | A | a |
| RIL135 | A | B | B | A | B | A | B | B | B | B | B | B | B | B | B | B | B | B | b |
| RIL136 | B | B | B | B | A | B | B | B | B | B | B | B | B | B | B | B | B | B | b |
| RIL137 | A | B | B | A | B | B | B | B | B | B | B | B | B | B | B | B | A | B | b |
| RIL138 | A | A | A | B | A | A | A | A | A | B | A | B | B | B | B | B | B | A | a |
| RIL139 | B | B | B | A | B | A | A | A | B | B | B | B | B | B | B | B | B | B | b |
| RIL140 | A | A | A | B | A | A | A | H | B | B | A | B | B | B | B | B | A | A | a |
| RIL141 | B | B | B | A | A | B | B | B | B | B | B | B | B | B | B | B | B | B | b |
| RIL142 | A | A | A | A | B | A | B | B | A | A | A | A | A | A | A | A | B | A | a |
| RIL143 | B | A | A | B | B | A | A | A | A | A | A | A | A | A | A | B | A | A | a |
| RIL144 | A | B | B | A | A | B | B | B | B | B | B | B | B | B | B | B | B | B | b |
| RIL146 | A | B | B | A | B | B | B | B | B | B | B | B | B | B | B | B | A | B | b |
| RIL147 | A | B | B | A | A | B | A | A | B | B | B | B | B | B | B | B | B | - | a |
| RIL148 | A | A | A | A | B | A | A | A | A | A | A | A | A | A | A | H | B | A | a |
| RIL149 | B | B | B | B | A | B | B | B | B | B | B | B | B | B | B | B | A | B | b |
| RIL150 | A | A | B | B | A | A | A | A | B | A | A | A | A | A | A | H | A | A | a |
| RIL151 | B | A | A | B | B | A | A | A | A | B | B | B | B | B | B | B | B | B | b |
| RIL152 | A | B | B | A | A | B | B | B | B | B | B | B | B | B | B | B | B | B | b |
| RIL153 | A | B | B | A | B | B | B | B | B | B | B | B | B | B | B | B | B | B | b |
| RIL154 | B | B | B | A | B | B | B | B | B | B | B | B | B | B | B | B | B | B | b |
| RIL155 | A | A | A | B | B | A | A | A | A | A | A | A | A | A | A | B | A | A | a |
| RIL156 | A | A | A | B | B | B | A | A | B | B | B | B | B | B | B | B | A | B | b |
| RIL157 | A | B | A | B | B | A | B | B | A | A | A | A | A | A | A | A | A | A | a |
| RIL158 | A | A | A | B | B | A | A | A | A | A | A | A | A | A | A | A | B | A | a |
| RIL159 | B | B | B | B | A | B | B | B | B | B | B | B | B | B | B | B | B | B | b |
| RIL160 | A | B | B | A | B | B | B | B | B | B | B | B | B | B | B | B | B | B | b |
| RIL161 | A | A | A | A | B | A | A | A | H | B | B | B | B | B | B | B | A | A | a |
| RIL162 | B | A | A | A | A | A | A | A | A | A | A | A | A | A | A | A | A | A | a |
| RIL163 | A | A | A | B | A | B | B | B | A | A | A | A | A | A | A | A | B | A | a |
| RIL164 | A | A | A | B | B | A | A | A | A | B | B | B | B | B | B | B | A | A | a |
| RIL165 | B | B | B | B | B | A | A | B | B | B | B | B | B | B | B | H | A | B | b |
| RIL166 | B | A | A | A | B | A | H | A | A | A | A | A | A | A | A | A | H | A | a |
| RIL167 | A | B | B | B | A | B | B | B | B | A | A | A | A | A | A | A | B | A | a |
| RIL168 | A | B | B | A | A | B | B | B | B | B | B | B | B | B | B | B | A | B | b |
| RIL170 | A | B | A | B | A | A | A | A | A | A | A | A | B | B | B | B | B | A | a |
| RIL171 | B | B | B | A | A | B | B | B | B | B | B | B | B | B | B | B | B | B | b |
| RIL172 | A | B | B | A | B | A | A | A | B | B | B | B | B | B | B | B | B | B | b |
| RIL174 | B | B | B | B | B | B | B | B | B | B | B | B | B | B | B | B | B | B | b |
| RIL175 | A | A | A | A | A | A | A | A | A | A | A | A | A | A | A | A | A | A | a |
| RIL176 | B | B | B | B | A | B | B | B | B | B | B | B | B | B | B | B | B | B | b |
| RIL177 | A | B | B | A | A | B | B | B | B | B | B | B | B | B | B | B | B | B | b |
| RIL178 | A | B | B | A | B | A | B | B | B | B | B | B | A | A | A | A | A | B | b |
| RIL179 | A | B | B | B | A | A | B | B | B | B | B | B | B | B | B | B | B | B | b |
| RIL180 | A | B | B | B | B | A | A | A | B | B | B | B | B | B | B | B | B | B | b |
| RIL181 | A | B | B | A | B | B | B | B | B | B | B | B | - | B | B | B | B | B | b |
| RIL183 | A | A | A | B | A | A | A | H | A | A | A | A | A | A | A | A | A | A | a |
| RIL186 | B | H | A | B | A | B | H | B | A | A | A | A | A | B | B | B | B | B | a |
| RIL187 | A | B | B | A | A | A | A | A | B | B | B | B | B | B | B | B | B | B | b |
| RIL188 | A | B | B | B | B | B | B | B | B | A | A | A | A | A | A | A | A | A | a |
| RIL189 | B | A | A | B | A | B | A | A | A | B | B | B | B | B | B | B | B | B | b |
| RIL190 | B | A | A | A | B | A | A | A | B | B | B | B | B | B | B | B | B | B | b |
| RIL191 | A | B | B | A | B | A | A | A | B | B | B | B | B | B | B | B | A | B | b |
| RIL192 | A | B | B | B | A | B | B | B | B | H | A | A | H | A | H | B | A | A | a |
| RIL194 | B | B | B | A | B | B | B | B | B | B | B | B | B | B | B | B | B | B | b |
| RIL195 | B | B | B | B | A | B | B | B | B | B | B | B | B | B | B | B | B | B | b |
| RIL196 | A | A | A | B | A | A | A | A | A | A | A | A | A | A | A | H | A | A | a |
| RIL197 | B | B | B | A | B | A | A | A | B | B | B | B | B | B | B | B | B | B | b |
| RIL198 | B | A | A | B | A | B | A | A | A | A | A | A | A | A | A | A | A | A | a |
| RIL200 | A | A | A | B | B | A | A | A | A | A | A | A | A | A | A | A | A | A | a |
| RIL201 | A | B | B | A | A | B | B | B | B | B | B | B | B | B | B | B | B | B | b |
| RIL203 | B | B | A | B | B | B | B | B | B | A | A | A | B | A | B | B | A | A | a |
| RIL204 | B | A | - | B | A | A | A | H | A | A | A | A | A | A | A | A | A | A | a |
| RIL205 | A | A | A | B | B | A | A | A | A | A | A | A | A | A | A | H | B | A | a |
| RIL206 | A | A | A | B | B | B | B | B | A | A | A | A | A | A | A | A | H | A | a |
| RIL207 | A | B | B | A | A | B | B | B | B | A | A | A | A | A | A | B | B | B | b |
| RIL208 | A | B | B | A | A | H | B | B | B | B | B | B | B | B | B | B | A | B | b |
| 222-09 |  |  |  |  |  |  |  |  |  |  |  |  |  |  |  |  |  | B | b |
| 222-10 |  |  |  |  |  |  |  |  |  |  |  |  |  |  |  |  |  | A | a |
| 222-12 |  |  |  |  |  |  |  |  |  |  |  |  |  |  |  |  |  | B | b |
| 222-13 |  |  |  |  |  |  |  |  |  |  |  |  |  |  |  |  |  | A | a |
| 222-15 |  |  |  |  |  |  |  |  |  |  |  |  |  |  |  |  |  | B | b |
| 222-22 |  |  |  |  |  |  |  |  |  |  |  |  |  |  |  |  |  | B | b |
| 222-23 |  |  |  |  |  |  |  |  |  |  |  |  |  |  |  |  |  | A | a |
| 223-02 |  |  |  |  |  |  |  |  |  |  |  |  |  |  |  |  |  | A | a |
| 223-12 |  |  |  |  |  |  |  |  |  |  |  |  |  |  |  |  |  | A | a |
| 223-13 |  |  |  |  |  |  |  |  |  |  |  |  |  |  |  |  |  | B | b |
| 223-14 |  |  |  |  |  |  |  |  |  |  |  |  |  |  |  |  |  | A | a |
| 223-15 |  |  |  |  |  |  |  |  |  |  |  |  |  |  |  |  |  | A | a |
| 223-18 |  |  |  |  |  |  |  |  |  |  |  |  |  |  |  |  |  | A | a |
| 223-20 |  |  |  |  |  |  |  |  |  |  |  |  |  |  |  |  |  | A | a |
| 223-22 |  |  |  |  |  |  |  |  |  |  |  |  |  |  |  |  |  | B | b |
| 223-24 |  |  |  |  |  |  |  |  |  |  |  |  |  |  |  |  |  | B | b |
| 223-25 |  |  |  |  |  |  |  |  |  |  |  |  |  |  |  |  |  | B | b |
| 223-26 |  |  |  |  |  |  |  |  |  |  |  |  |  |  |  |  |  | B | b |
| 223-27 |  |  |  |  |  |  |  |  |  |  |  |  |  |  |  |  |  | A | a |
| 224-01 |  |  |  |  |  |  |  |  |  |  |  |  |  |  |  |  |  | B | b |
| 224-06 |  |  |  |  |  |  |  |  |  |  |  |  |  |  |  |  |  | A | a |
| 224-13 |  |  |  |  |  |  |  |  |  |  |  |  |  |  |  |  |  | A | a |
| 224-21 |  |  |  |  |  |  |  |  |  |  |  |  |  |  |  |  |  | A | a |
| 224-23 |  |  |  |  |  |  |  |  |  |  |  |  |  |  |  |  |  | A | a |
| 225-03 |  |  |  |  |  |  |  |  |  |  |  |  |  |  |  |  |  | A | a |
| 225-10 |  |  |  |  |  |  |  |  |  |  |  |  |  |  |  |  |  | A | a |
| 225-11 |  |  |  |  |  |  |  |  |  |  |  |  |  |  |  |  |  | A | a |
| 225-16 |  |  |  |  |  |  |  |  |  |  |  |  |  |  |  |  |  | B | b |
| 225-20 |  |  |  |  |  |  |  |  |  |  |  |  |  |  |  |  |  | B | b |
| 225-21 |  |  |  |  |  |  |  |  |  |  |  |  |  |  |  |  |  | B | b |
| 225-23 |  |  |  |  |  |  |  |  |  |  |  |  |  |  |  |  |  | A | a |
| 225-24 |  |  |  |  |  |  |  |  |  |  |  |  |  |  |  |  |  | A | a |
| 225-25 |  |  |  |  |  |  |  |  |  |  |  |  |  |  |  |  |  | A | a |
| 225-27 |  |  |  |  |  |  |  |  |  |  |  |  |  |  |  |  |  | B | b |
| 225-28 |  |  |  |  |  |  |  |  |  |  |  |  |  |  |  |  |  | **B** | **a** |
| 226-03 |  |  |  |  |  |  |  |  |  |  |  |  |  |  |  |  |  | B | b |
| 226-07 |  |  |  |  |  |  |  |  |  |  |  |  |  |  |  |  |  | B | b |
| 226-10 |  |  |  |  |  |  |  |  |  |  |  |  |  |  |  |  |  | A | a |
| 226-11 |  |  |  |  |  |  |  |  |  |  |  |  |  |  |  |  |  | B | b |
| 226-13 |  |  |  |  |  |  |  |  |  |  |  |  |  |  |  |  |  | B | b |
| 226-14 |  |  |  |  |  |  |  |  |  |  |  |  |  |  |  |  |  | A | a |
| 226-19 |  |  |  |  |  |  |  |  |  |  |  |  |  |  |  |  |  | B | b |
| 226-24 |  |  |  |  |  |  |  |  |  |  |  |  |  |  |  |  |  | B | b |
| 226-25 |  |  |  |  |  |  |  |  |  |  |  |  |  |  |  |  |  | A | a |
| 227-02 |  |  |  |  |  |  |  |  |  |  |  |  |  |  |  |  |  | A | a |
| 227-04 |  |  |  |  |  |  |  |  |  |  |  |  |  |  |  |  |  | B | b |
| 227-05 |  |  |  |  |  |  |  |  |  |  |  |  |  |  |  |  |  | B | b |
| 227-10 |  |  |  |  |  |  |  |  |  |  |  |  |  |  |  |  |  | A | a |
| 227-11 |  |  |  |  |  |  |  |  |  |  |  |  |  |  |  |  |  | A | a |
| 227-16 |  |  |  |  |  |  |  |  |  |  |  |  |  |  |  |  |  | B | b |
| 227-19 |  |  |  |  |  |  |  |  |  |  |  |  |  |  |  |  |  | A | a |
| 227-20 |  |  |  |  |  |  |  |  |  |  |  |  |  |  |  |  |  | B | b |
| 227-21 |  |  |  |  |  |  |  |  |  |  |  |  |  |  |  |  |  | A | a |
| 227-22 |  |  |  |  |  |  |  |  |  |  |  |  |  |  |  |  |  | B | b |
| 227-23 |  |  |  |  |  |  |  |  |  |  |  |  |  |  |  |  |  | A | a |
| 227-24 |  |  |  |  |  |  |  |  |  |  |  |  |  |  |  |  |  | B | b |
| 227-25 |  |  |  |  |  |  |  |  |  |  |  |  |  |  |  |  |  | B | b |
| 227-26 |  |  |  |  |  |  |  |  |  |  |  |  |  |  |  |  |  | A | a |
| 227-27 |  |  |  |  |  |  |  |  |  |  |  |  |  |  |  |  |  | B | b |
| 227-28 |  |  |  |  |  |  |  |  |  |  |  |  |  |  |  |  |  | A | a |
| 227-29 |  |  |  |  |  |  |  |  |  |  |  |  |  |  |  |  |  | B | b |
| 227-30 |  |  |  |  |  |  |  |  |  |  |  |  |  |  |  |  |  | A | a |
| 228-01 |  |  |  |  |  |  |  |  |  |  |  |  |  |  |  |  |  | A | a |
| 228-03 |  |  |  |  |  |  |  |  |  |  |  |  |  |  |  |  |  | A | a |
| 228-04 |  |  |  |  |  |  |  |  |  |  |  |  |  |  |  |  |  | B | b |
| 228-05 |  |  |  |  |  |  |  |  |  |  |  |  |  |  |  |  |  | A | a |
| 228-06 |  |  |  |  |  |  |  |  |  |  |  |  |  |  |  |  |  | B | b |
| 228-07 |  |  |  |  |  |  |  |  |  |  |  |  |  |  |  |  |  | A | a |
| 228-08 |  |  |  |  |  |  |  |  |  |  |  |  |  |  |  |  |  | B | b |
| 228-09 |  |  |  |  |  |  |  |  |  |  |  |  |  |  |  |  |  | - | b |
| 228-10 |  |  |  |  |  |  |  |  |  |  |  |  |  |  |  |  |  | B | b |
| 228-11 |  |  |  |  |  |  |  |  |  |  |  |  |  |  |  |  |  | B | b |
| 228-12 |  |  |  |  |  |  |  |  |  |  |  |  |  |  |  |  |  | A | a |
| 228-13 |  |  |  |  |  |  |  |  |  |  |  |  |  |  |  |  |  | B | b |
| 228-15 |  |  |  |  |  |  |  |  |  |  |  |  |  |  |  |  |  | B | b |
| 228-19 |  |  |  |  |  |  |  |  |  |  |  |  |  |  |  |  |  | A | a |
| 228-20 |  |  |  |  |  |  |  |  |  |  |  |  |  |  |  |  |  | B | b |
| 228-21 |  |  |  |  |  |  |  |  |  |  |  |  |  |  |  |  |  | B | b |
| 228-22 |  |  |  |  |  |  |  |  |  |  |  |  |  |  |  |  |  | B | b |
| 228-23 |  |  |  |  |  |  |  |  |  |  |  |  |  |  |  |  |  | B | b |
| 228-24 |  |  |  |  |  |  |  |  |  |  |  |  |  |  |  |  |  | A | a |
| 228-25 |  |  |  |  |  |  |  |  |  |  |  |  |  |  |  |  |  | B | b |
| 228-26 |  |  |  |  |  |  |  |  |  |  |  |  |  |  |  |  |  | A | a |
| 228-27 |  |  |  |  |  |  |  |  |  |  |  |  |  |  |  |  |  | B | b |
| 228-28 |  |  |  |  |  |  |  |  |  |  |  |  |  |  |  |  |  | B | b |
| 228-29 |  |  |  |  |  |  |  |  |  |  |  |  |  |  |  |  |  | A | a |
| 229-01 |  |  |  |  |  |  |  |  |  |  |  |  |  |  |  |  |  | B | b |
| 229-02 |  |  |  |  |  |  |  |  |  |  |  |  |  |  |  |  |  | A | a |
| 229-04 |  |  |  |  |  |  |  |  |  |  |  |  |  |  |  |  |  | B | b |
| 229-05 |  |  |  |  |  |  |  |  |  |  |  |  |  |  |  |  |  | A | a |
| 229-06 |  |  |  |  |  |  |  |  |  |  |  |  |  |  |  |  |  | B | b |
| 229-07 |  |  |  |  |  |  |  |  |  |  |  |  |  |  |  |  |  | B | b |
| 229-08 |  |  |  |  |  |  |  |  |  |  |  |  |  |  |  |  |  | B | b |
| 229-09 |  |  |  |  |  |  |  |  |  |  |  |  |  |  |  |  |  | A | a |
| 229-10 |  |  |  |  |  |  |  |  |  |  |  |  |  |  |  |  |  | A | a |
| 229-11 |  |  |  |  |  |  |  |  |  |  |  |  |  |  |  |  |  | A | a |
| 229-16 |  |  |  |  |  |  |  |  |  |  |  |  |  |  |  |  |  | A | a |
| 229-18 |  |  |  |  |  |  |  |  |  |  |  |  |  |  |  |  |  | A | a |
| 229-20 |  |  |  |  |  |  |  |  |  |  |  |  |  |  |  |  |  | A | a |
| 229-22 |  |  |  |  |  |  |  |  |  |  |  |  |  |  |  |  |  | A | a |
