# Supplementary material for: Fine-mapping of a putative glutathione S-transferase (GST) gene responsible for yellow seed colour in flax (Linum usitatissimum)
Source: BMC Res Notes. 2022 Feb 20;15:72. doi: 10.1186/s13104-022-05964-x (PMC8859895; doi:10.1186/s13104-022-05964-x)
Supplement: Supplementary file 5 — Additional file 5: Data S2. Sequences of Lus10019895 for CDC Bethune and S95407. [file 13104_2022_5964_MOESM5_ESM.docx]

**Data S2**: Sequences of *Lus10019895* for CDC Bethune and S95407

LOCUS Beth_Lus10019895 1421 bp DNA linear UNA 08-NOV-2020

DEFINITION .

ACCESSION geneious|urn:local:

VERSION geneious|urn:local:.:1a-cmhg2sm:0

KEYWORDS .

SOURCE

ORGANISM .

FEATURES Location/Qualifiers

gene <1..1421

/standard_name="gene"

misc_feature <1..1421

/standard_name="transcript"

variation 57

/standard_name="SNP"

CDS 78..88

/codon_start=1

/standard_name="CDS"

intron 89..189

/standard_name="intron"

variation 170

/standard_name="SNP"

CDS 190..343

/codon_start=2

/standard_name="CDS"

variation 275..277

/standard_name="NS-SNP T->I"

variation 311..313

/standard_name="NS-SNP A->S"

intron 344..448

/standard_name="intron"

variation 403

/standard_name="SNP"

variation 446

/standard_name="SNP"

CDS 449..631

/codon_start=1

/standard_name="CDS"

intron 632..714

/standard_name="intron"

CDS 715..817

/codon_start=1

/standard_name="CDS"

variation 718..720

/standard_name="NS-SNP T->A"

variation 733..735

/standard_name="NS-SNP F->Y"

variation 771

/standard_name="SNP"

intron 818..906

/standard_name="intron"

CDS 907..986

/codon_start=3

/standard_name="CDS"

intron 987..1058

/standard_name="intron"

variation 1051

/standard_name="SNP"

CDS 1059..1247

/codon_start=1

/standard_name="CDS"

variation 1133

/standard_name="SNP"

intron 1248..1345

/standard_name="intron"

CDS 1346..1360

/codon_start=1

/standard_name="CDS"

misc_feature 1358..1360

/standard_name="stop_codon"

ORIGIN

1 caatagccac caagtttcag aatttggtac ctgaaagaca cagaagtagg aagctccgtt

61 ctaattgcag cttagacatg gcggcagagt aagaactttg caaagaacat tatgaaagtt

121 ttttttattg ttcctttttt tagccaaatc atcaattgtt tcaccattgc gtgttatgtt

181 ggttggaagt tcgtatatat ccaacgccag agagtctcta cctccgtcac tggattccgc

241 cagccacgcg ccgcctccct ttaacggaac cgccaccgcc agattgtaca cgagttacag

301 aagcccattt gctcagcgcg cctggattgc gaggaattgc aaggtcgacg acctgctaat

361 tttttttact ggtttcatca tatatcactt cttctccctt gctcaaacta atccatgaaa

421 agtatatata ctgtttgttt tttcgtaggg tttaaaggac aagatccagc tggtcccgat

481 aaaccttgaa gacaagccgt cctggtacaa gcaagtaaac ccacaaggaa aggtgccagc

541 tttggaacac aacggtaaaa tcatcgcgga aagcctcgat atattacgct atgtcgacac

601 caatttcgac ggcccaccac tcttcccaga cgtaagattc aatcgtattc caaacataca

661 tcagaagcga tacaataagt tgtgacccct tttggaattt ggtcgttaaa acaggatact

721 gaaaggaaaa gttttgctga ggaattgctg tcctactctg ataccttcac tgagatgatt

781 ctcaactctt ttaaaggaga aacagtaaga gaagccggta atatttgtat attctggatt

841 actacttctt tattagtttt cttggctgca tcaaacgggc tgagacgata aattgtttgc

901 tgacagatcc tgcatttgat gtggtggaag ctgcccttgg gaagttcaaa gacggaccct

961 ttttccttgg cagacagttc agcttggtca caacactatc tctaatcagt cttttactgt

1021 gtgaagaatg gcgtttaatt tgcactctgt ctcttcaggt ggacatagct tacattccgt

1081 tcgttgaaag agcagagata ttcctgcctg cagcttggaa ctacgatatc actggtggaa

1141 ggccacaact tgctgcttgg attcaggaaa tgaagagggt tgatggggtc aaggaaacga

1201 agagcgattc cagagaggtg gtcggcttct acaagaatcg cttcttggta ttattattac

1261 tctgtgtgtg cctgatgatt ctgtttatag ttagttttac aatttgcttg gtaacgattt

1321 catcaccaaa ctcattattt tgcaggcaca ggatcagtga ccgctaaggg gcaaattttc

1381 atgttgtaga ttccttgttc ttgcagatta cgaacgaaat g

//

LOCUS S95407_Lus10019895 1421 bp DNA linear UNA 08-NOV-2020

DEFINITION .

ACCESSION geneious|urn:local:

VERSION geneious|urn:local:.:1a-cmhg2sm:1

KEYWORDS .

SOURCE

ORGANISM .

FEATURES Location/Qualifiers

gene <1..1421

/standard_name="gene"

misc_feature <1..1421

/standard_name="transcript"

variation 57

/standard_name="SNP"

CDS 78..88

/codon_start=1

/standard_name="CDS"

intron 89..189

/standard_name="intron"

variation 170

/standard_name="SNP"

CDS 190..343

/codon_start=2

/standard_name="CDS"

variation 275..277

/standard_name="NS-SNP T->I"

variation 311..313

/standard_name="NS-SNP A->S"

intron 344..448

/standard_name="intron"

variation 403

/standard_name="SNP"

variation 446

/standard_name="SNP"

CDS 449..631

/codon_start=1

/standard_name="CDS"

intron 632..714

/standard_name="intron"

CDS 715..817

/codon_start=1

/standard_name="CDS"

variation 718..720

/standard_name="NS-SNP T->A"

variation 733..735

/standard_name="NS-SNP F->Y"

variation 771

/standard_name="SNP"

intron 818..906

/standard_name="intron"

CDS 907..986

/codon_start=3

/standard_name="CDS"

intron 987..1058

/standard_name="intron"

variation 1051

/standard_name="SNP"

CDS 1059..1247

/codon_start=1

/standard_name="CDS"

variation 1133

/standard_name="SNP"

intron 1248..1345

/standard_name="intron"

CDS 1346..1360

/codon_start=1

/standard_name="CDS"

misc_feature 1358..1360

/standard_name="stop_codon"

ORIGIN

1 caatagccac caagtttcag aatttggtac ctgaaagaca cagaagtagg aagctcggtt

61 ctaattgcag cttagacatg gcggcagagt aagaactttg caaagaacat tatgaaagtt

121 ttttttattg ttcctttttt tagccaaatc atcaattgtt tcaccattgt gtgttatgtt

181 ggttggaagt tcgtatatat ccaacgccag agagtctcta cctccgtcac tggattccgc

241 cagccacgcg ccgcctccct ttaacggaac cgccatcgcc agattgtaca cgagttacag

301 aagcccattt tctcagcgcg cctggattgc gaggaattgc aaggtcgacg acctgctaat

361 tttttttact ggtttcatca tatatcactt cttctccctt gcgcaaacta atccatgaaa

421 agtatatata ctgtttgttt tttcgaaggg tttaaaggac aagatccagc tggtcccgat

481 aaaccttgaa gacaagccgt cctggtacaa gcaagtaaac ccacaaggaa aggtgccagc

541 tttggaacac aacggtaaaa tcatcgcgga aagcctcgat atattacgct atgtcgacac

601 caatttcgac ggcccaccac tcttcccaga cgtaagattc aatcgtattc caaacataca

661 tcagaagcga tacaataagt tgtgacccct tttggaattt ggtcgttaaa acaggatgct

721 gaaaggaaaa gttatgctga ggaattgctg tcctactctg ataccttcac cgagatgatt

781 ctcaactctt ttaaaggaga aacagtaaga gaagccggta atatttgtat attctggatt

841 actacttctt tattagtttt cttggctgca tcaaacgggc tgagacgata aattgtttgc

901 tgacagatcc tgcatttgat gtggtggaag ctgcccttgg gaagttcaaa gacggaccct

961 ttttccttgg cagacagttc agcttggtca caacactatc tctaatcagt cttttactgt

1021 gtgaagaatg gcgtttaatt tgcactctgt ttcttcaggt ggacatagct tacattccgt

1081 tcgttgaaag agcagagata ttcctgcctg cagcttggaa ctacgatatc acgggtggaa

1141 ggccacaact tgctgcttgg attcaggaaa tgaagagggt tgatggggtc aaggaaacga

1201 agagcgattc cagagaggtg gtcggcttct acaagaatcg cttcttggta ttattattac

1261 tctgtgtgtg cctgatgatt ctgtttatag ttagttttac aatttgcttg gtaacgattt

1321 catcaccaaa ctcattattt tgcaggcaca ggatcagtga ccgctaaggg gcaaattttc

1381 atgttgtaga ttccttgttc ttgcagatta cgaacgaaat g
